# Financial management roles of nurse managers in selected public hospitals in KwaZulu-Natal province, South Africa

**DOI:** 10.4102/phcfm.v11i1.1981

**Published:** 2019-09-18

**Authors:** Nellie Naranjee, Thembelihle S.P. Ngxongo, Maureen N. Sibiya

**Affiliations:** 1 KwaZulu Natal College of Nursing, Pietermaritzburg, Durban, South Africa; 2 Department of Nursing, Durban University of Technology, Durban, South Africa; 3 Faculty of Health Sciences, Durban University of Technology, Durban, South Africa

**Keywords:** Competency, Financial management framework, Nurse manager, Public hospitals, Constructivist grounded theory, Naturalistic paradigm

## Abstract

**Background:**

The public health sector in South Africa has been facing severe financial cutbacks and financial constraints in recent times. The nurse manager (NM) is faced with the task of managing and reducing expenditure in the nursing sector without compromising the quality care. This requires skills and understanding of financial management.

**Aim:**

This study aimed to explore the financial management roles of NMs and to identify financial management development needs necessary for NMs’ practice.

**Setting:**

The study was conducted in KwaZulu-Natal. A total of eight hospitals from the five health districts were included.

**Methods:**

The study used the naturalistic paradigm with a constructivist grounded theory approach. Interviews were used to initially gather data from six NMs who were purposively selected. Theoretical sampling was used to further recruit financial managers, chief executive officers, assistant nurse managers and operational managers. The final sample consisted of 18 participants.

**Results:**

Financial management of the hospitals is the primary function of the financial managers and the chief executive officers. However, the role of NMs extends to the performance and participation in various activities relating to the financial functioning of the hospital. These include financial planning, financial monitoring, financial decision-making and financial control.

**Conclusion:**

Nurse managers have a financial management function in public health care organisations but lack the necessary skills, knowledge and competencies to function in this role and require additional training. Recommendations included that a competency framework be developed to improve the financial management competencies of NMs.

## Introduction

In the public South African health care sector, the nurse manager (NM) is responsible for and oversees the nursing section of the health care organisation. In recent times, South Africa’s public health sector has been struck with reduced budget allocations and economic constraints, especially in the curative and tertiary sectors.^1^ This has called for NMs to manage the nursing departments with limited funding and find new ways to overcome and manage the financial challenges in the public sector. In order to respond to these challenges, NMs should have the correct and relevant managerial and financial competencies. The traditional management skill of NMs of which financial management is inadequate is no longer sufficient in the demanding public health care environment. An increased awareness of financial competence is emerging among nurses, but many health care organisations still find that financial management is one of the main shortcomings of the NMs.^2^ Financial skills of NMs are weak resulting in them and other nurses not being able to understand finances and not taking responsibility for the financial functioning of the nursing departments.^3^

The role of NM has changed significantly compared to 25 years ago when most NMs trained. The business of the hospital’s finances was traditionally beyond the control of the professional nurse. Today, every nurse needs to understand that hospitals in both the public and private sector need finances to function. In the public sector, financial management is not considered as a critical function of the NM when considering employment or promotion. Yet, the NM is expected to understand and manage the financial side of the nursing sector on assuming this role.^4^ An understanding of health care costs gives them insight into leadership and management challenges facing the institution and can improve patient care accordingly. Therefore, nurses need to be educated on the roles that they play in managing the hospital’s finances.^5^

Most NMs gain the financial knowledge and skills mainly through experience, trial and error. Waxman finds that NMs do not receive adequate training in this subject, even though they are held accountable for the budget in their areas, and recommends for more education and training to assist NMs in acquiring financial management skills.^6^ Rundio attests to that financial management is not addressed adequately in nursing curricula as the focus is on clinical rather than financial skills.^7^

Even when nurses are promoted into management, they do not always get training or the necessary work experience in managing finances.^8^ It is imperative that NMs are prepared for their financial management competencies and skills so that they will be able to manage expenditure and contain costs within their departments. The development of financial management skills and competencies can assist NMs to better manage the current health care situation.

### Aim of the study

The aim of the study was to explore the financial management roles of NMs in their current work environments.

## Research methods and design

### Theoretical frameworks

This type of study required several perspectives on the financial management competencies and the financial management developmental needs of the research participants. For this reason, the foundational paradigm for this study was based on Lincoln and Guba’s (1985) theoretical elements of the naturalistic inquiry.^9^ The naturalistic paradigm offered the correct method for gaining the detailed information necessary to understand the financial management roles and practices of NMs, as it is experienced in the hospital settings. Such an understanding was critical during the data analysis phase as it helped with the development of a financial management competency framework for NMs (Figure 2). Grounded theory (GT) is described as a flexible, qualitative, inductive approach which is comprehensive, integrated and highly structured and follows a set of procedures based on the constant comparative method.^10^ Charmaz’s (2006) constructivist GT approach was used. Constructivist grounded theorists seek to understand the difference and variation among research participants and to co-construct meaning with them.^11^

### Setting

The study was conducted in KwaZulu-Natal, which is one of the nine provinces in South Africa. A total of 32 district and regional hospitals in all 11 health districts in KwaZulu-Natal were invited to participate in the study. Eight public hospitals in five health districts participated in the study. The hospitals included four urban and four rural hospitals. There were three regional and five district hospitals that participated in the study as illustrated in Table 1.

**TABLE 1:** Health districts and participating hospitals.

Health district	Hospital and code	Regional or district	Rural or urban
Ugu	Hospital 1 (H 1)	District	Rural
	Hospital 2 (H 2)	District	Rural
EThekwini	Hospital 3 (H 3)	Regional	Urban
	Hospital 4 (H 4)	Regional	Urban
	Hospital 5 (H 5)	District	Urban
UThukela	Hospital 6 (H 6)	District	Rural
UMgungundlovu	Hospital 7 (H 7)	Regional	Urban
Sisonke	Hospital 8 (H 8)	District	Rural

Total health districts = 5.

### Study population and sampling strategy

The total number of participants was 18. A total of six NMs were initially recruited using the purposive sampling method. Three NMs were from rural district hospitals and three were from urban regional hospitals. Data analysis began immediately after each interview consistent with the GT approach. As data emerged, it became apparent that there were other role players in the hospital that were also involved in financial management together with the NM. There was value in including these participants as they could provide an added perspective to the study in view of their experiences. In keeping with GT, theoretical sampling proceeded to include three financial managers (FMs), five operational managers (OMs), two assistant nursing managers (ANMs) and two CEOs of the hospitals. Theoretical sampling involves decisions about what data to collect next and where to find these data to develop an emerging theory optimally. The basic question in theoretical sampling is what groups or subgroups should the researcher turn to next.^12^ The participants and their respective hospitals are presented in Table 2.

**TABLE 2:** Hospitals and participants.

Health district	Hospital and code	Number of interviews and participant				
		NM	ANM	OM	FM	CEO
Ugu	Hospital 1 (H 1)	1	-	-	1	-
	Hospital 2 (H 2)	1	-	-	1	-
EThekwini	Hospital 3 (H 3)	1	-	-	-	-
	Hospital 4 (H 4)	1	1	1	-	-
	Hospital 5 (H 5)	-	-	2	-	1
UThukela	Hospital 6 (H 6)	1	-	-	1	-
UMgungundlovu	Hospital 7 (H 7)	1	1	2	-	-
Sisonke	Hospital 8 (H 8)	-	-	-	-	1
**Total**	**8**	**6**	**2**	**5**	**3**	**2**

Total Health districts = 5.

FM, financial manager; NM, nurse manager; ANM, assistant nursing managers; OM, operational managers.

### Data collection

Semi- structured interviews were used to collect data. The interviews lasted between 1 hour and 3 hours and were conducted in participant’s offices or in the boardroom of the hospitals. The interviews were conducted by the researcher and were all audio-recorded.

### Data analysis

Data collection and analysis were performed simultaneously consistent with the GT method. Interviews were transcribed verbatim from the audio-recorders within 24 h and replayed to ensure accurate transcriptions. The audio-recorded transcriptions were further reviewed, identifying wording which correlated with the research questions. Memo writing and comparative analysis were performed throughout the study and also assisted the process of open, focused and theoretical coding as suggested by Charmaz.^13^ Eight major themes and several sub-themes emerged from the findings (Table 3).

**TABLE 3:** Themes and sub-themes.

Research objective	Themes	Sub-themes
1. Explore the current roles of the NM with regard to financial management practice in public health care organisations	Theme 1: Financial planning	Strategic planning
		Operational planning
		Procurement planning
		Human resource planning
		Budget planning
	Theme 2: Financial monitoring	Cash flow monitoring
		Analysis and interpretation of financial statements
	Theme 3: Financial decision-making	Approval of contracts and tenders
		Acting in the CEO role
		Financial legislative framework
	Theme 4: Financial control	Assets and resources control
		Expenditure control
2. Determine the financial management competencies that NMs in public health care organisations currently possess.	Theme 5: Inadequate financial management competencies	Poor involvement in financial management activities
		Lack of in- service and financial management training for current role
		Experiential learning
3. Explore the training and educational qualifications NMs have with respect to financial management	Theme 6: Educational preparation for financial management in nursing programmes	Formal educational preparation for financial management in nursing programmes
		Inadequate financial management training in basic and post-basic nursing programmes
	Theme 7: Guidance and training for financial management role	Informal financial management training for current role
		Poor support from Department of Health for current financial management role
		Peer support and mentoring for the financial management role
4. Establish the essential financial management development needs of NMs in public health care organisations	Theme 8: Financial management competency development	Compulsory financial management training for NMs in the public sector before being promoted or after promotion
		Training in budget and budgetary concepts
		Need for NMs to understand financial reports
		Training in cost centre management
		Training in financial management legislation

NM, nurse manager.

### Ethical considerations

Ethical clearance was granted by the research committee of the study institution. Permission was granted overall by the KwaZulu-Natal Department of Health Research and Knowledge Management to conduct the study at all 32 hospitals in all 11 health districts. Before commencement of the study, a letter was sent to all NMs, informing them about the study and the benefits and risks. Once the other participants, namely ANMs, OMs, FMs and CEOs, were identified, letters were sent to them providing information on the study and requesting their permission to participate. The participants were informed that they could withdraw from the study at any time with no penalties. Written consent was signed by each participant prior to the commencement of each interview. Anonymity and confidentiality were maintained as names of study sites and participants were not disclosed when the findings were published.

## Results

**FIGURE 1:**
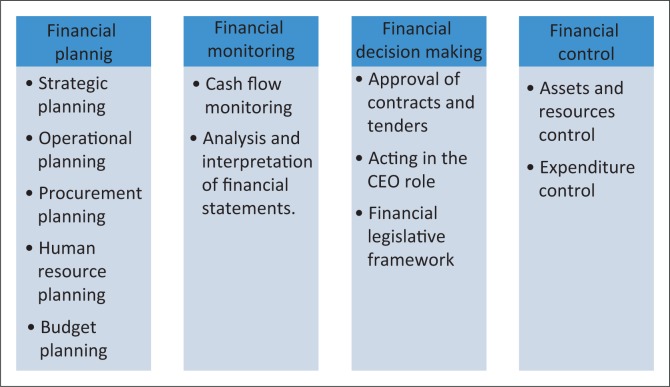
Financial management roles and activities of nurse managers.

Four major themes emerged from the exploration of the financial management roles and responsibilities of the NMs, as well as the financial activities that they practice in their current positions, namely financial planning, financial monitoring, financial decision-making and financial control (Figure 1).

*Financial planning* was a major theme identified with respect to the financial management role of the NM. Strategic management was part of the financial planning process:

‘We attend strategic management workshops and we participate in the planning for the next 2 years based on the budget we have.’ (Participant 10, male, operational manager)

Another role of the NM included operational or business planning:

‘Financial management activities of the NM start with operational plans. We convert the operational plans to budget. Whatever is financed is based on the planning by NMs. They are responsible for drawing up business plans.’ (Participant 15, male, financial manager)

Procurement planning is another financial planning function:

‘Every year there is a procurement plan that we have to draw up. The OMs and ANMs do the plan together and then I sit with each component before the plan is forwarded to the FM.’ (Participant 1, female, nurse manager)

Human resource planning is performed by the NM to ensure that there is the appropriate number of nursing staff to cater for patient care needs:

‘When we talk about financial management, within my component I am responsible for the management of human resources for nursing. We have to weigh the posts as it is linked to the budget.’ (Participant 6, female, nurse manager)

Part of financial planning also included participation in budget development:

‘There is involvement in the budget but in a very subtle way. You are not asked to say how much you want. But right now OMs in each department are asked to submit their procurement plans and operational plan. So there is some involvement and they do have an input eventually into the budget.’ (Participant 5, male, nurse manager)

*Financial monitoring* was the second theme that was identified with respect to the financial management role of the NM.

NMs are part of the cash flow committee where they monitor and account for the financial performance of the nursing departments:

‘In terms of the expenditure, we have to monitor cost drivers. That is part of the cash flow. The whole team is responsible. The NM is part of all this in terms of monitoring and decision-making.’ (Participant 16, male, financial manager)

Financial statements are analysed by NMs to validate expenditure in the different departments based on the information presented in this statement:

‘We do get those reports and at every cash flow that we review, we go through a very short little condensed report just to make sure we are online. We look at what we got to spend, how much we spent and how much we overspent.’ (Participant 3, female, nurse manager)

The third theme related to the financial roles of the NMs was that of *financial decision-making.*

NMs participate in bid committees and decisions are made in terms of how contracts and tenders are awarded in the public sector:

‘The NMs are part of the decision makers in terms of what’s needed in the hospital and which is going to be used for the purpose of giving proper nursing care for the patient.’ (Participant 18, female, chief executive officer)

NMs often take charge of the hospital in the absence of the CEO and this involves a large amount of decision-making which often has financial implications:

‘At times we act as the CEO. You just sign not understanding fully what are you signing and also the implications. We need detailed explanations but in a simplified manner for NMs, because at the end of the day, you act as a CEO.’ (Participant 2, female, nurse manager)

Theme four was that of *financial control*.

A major role of the NM was asset and resource control:

‘On a daily basis we deal with HR [*human resource*], equipment and consumables. If you think about HR management, management of resources, equipment, time management, these are also part of financial management.’ (Participant 1, female, nurse manager)

Prevention of wastage and control was the function of all nurses:

‘The bulk of the staff in the institution, 80% is made up of nurses. So if expenditure control is treated and understood by nursing, you have half won the battle. If you manage things well, you can actually spread your money and it can go far. Where you are having waste, you are in control. If the NM understands finances, then she can control.’ (Participant 6, female, nurse manager)

## Discussion

### Key findings

**FIGURE 2:**
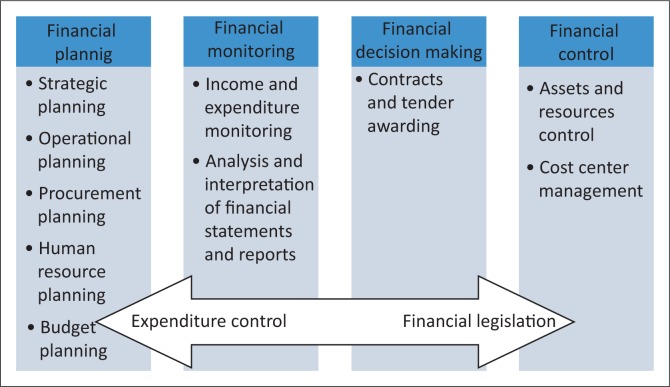
Components of a financial management competency framework for nurse managers.

The key findings which were used to develop a financial management competency framework are summarised in Figure 2 and are based on the themes and related sub-themes that emerged in relation to the objectives of the study.

According to Sherman,^14^ the role of the NM has changed radically over the past decade in that the clinical and interpersonal skills that led to the selection of many managers in the first place are simply not enough to ensure good performance. The NM role has a strong business component that needs to be both acknowledged and supported. Sherman^14^ acknowledges that on many levels, the success of a health care organisation is extremely dependent on the performance of NMs. A concern raised by Sherman^14^ was that considerable changes in the role have received little acknowledgement by most health systems and managers often receive little in the way of leadership development and NMs struggle in a role for which they are not prepared. Taking this into consideration, it was essential to firstly establish what financial management activities NMs were carrying out in their daily practice. The four major themes that emerged with regard to the current roles of the NM in financial management practice in public health care organisations included financial planning, financial monitoring, financial decision-making and financial control.

### Theme 1: Financial planning

Contrary to the view of many nurses that financial management belongs to the financial department, NMs were very clear in defining what exactly they do in terms of their financial management roles.^15^ Participation in strategic planning, procurement planning, operational planning, human resource planning and budget planning was identified as the financial planning role of NMs. They identified their generic management function of planning and aligned their functions and participation in each of the preceding activities in the context of financial planning. The findings reveal that generally, managers at all levels in public hospitals are involved to some extent in financial planning.

The NMs are not directly involved in the financial management of the hospitals, which is primarily the function of the Chief Financial Officer and the CEO. Nevertheless, they play a critical role by performing various activities that contribute to the financial functioning of the hospital. Fiegenbaum^16^ agrees that NMs play a critical role in hospital operations. There is an interdependent function as the CFO and CEO depend on the information provided by the NMs in order to plan finances and the budget. In the public hospitals, managers at all levels in the hospital such as the OMs, SCM managers and human resource (HR) managers are involved in these financial planning activities. Hartley^17^ found that poor knowledge about budgeting and finances was a challenge for NMs who were required to manage their departments in a cost-effective manner. The nursing budget comprised more than half of the total hospital budget and pressure is on NMs to increase efficiency and effectiveness.^17^

Muller et al.^18^ found that financial management is directly related to the strategic management process of an organisation. The findings of this study revealed that participants were able to correlate the drawing up of the operational plans and procurement plans which were based on the strategic plan. Participants were also able to identify how these plans fit into the strategic plan and the big plan for the hospital which was eventually integrated into the provincial strategic plan. They did not encounter any problem in formulating strategic plans. Similarly, Asamani et al.^19^ confirm top-level managers, including nurse executives or hospital matrons, usually undertake strategic planning to establish long-term goals to reinforce the hospital’s mission.

It was noted in the findings of this study that NMs and OMs prepare the procurement plan according to the needs of the department. The NMs and OMs indicated that they requested or ordered items based on their departmental needs. According to Fourie,^20^ public managers play a crucial role in the procurement of goods and services as applicants or buyers. In the public service, the National Treasury, accounting officers, CFOs, provincial treasury and bid committees are responsible for SCM. Emerging from this context, participants in this study verbalised inclusion and consultation with the interdisciplinary health team when drawing up the procurement plan. Nurse managers, though not directly involved in the drawing up of the budget, contribute by the way of procurement plans.

Booyens et al.^21^ assert that human resources management is the most important and challenging function of management because the outcomes of a unit or organisation depend largely on the staff. One of the central responsibilities of the NM as noted in this study was that of human resource planning. Nurse managers indicated that their roles in financial planning included ensuring that there is the appropriate number and mix of nursing to match actual or projected patient care needs. Finkler et al.^22^ find that the largest part of the operating budgets for nursing departments and organisations consists of personnel costs. The researcher noted that the NMs found HR planning to be a very stressful part of their financial planning role. This was because of the number of posts being restricted by the Department of Health and the high number of vacancies in the hospitals. It was very difficult to staff the departments adequately to ensure reasonable standard of care. Shirey’s^23^ study revealed similar findings where human resources and staffing were the biggest cause of stress for NMs. The inability to procure the necessary human resources needed to operate their units was cited as most burdening for NMs.

Booyens et al.^21^ describe a budget as a detailed plan for the acquisition and use of financial and other resources over a specific period of time. It is an amalgamation of expenditure plans and revenue origination plans for the coming year. It was evident that the general perception among NMs in this study was that although they were not fully involved in the budget planning and development, they were involved to a certain level. The findings reveal that NMs do not actually draw up a budget but perform and coordinate various activities that contribute to the formulation of the budget. Nurse managers’ participation in budget development is limited to the input provided in the procurement plans and operational plans submitted by the various nursing departments. One of the shortcomings of the NMs as noted in this study was the lack of knowledge regarding the budget process and how it is drawn up. Participants expressed that the budget was drawn up on historical basis and the previous orders from the stores department. The findings are confirmed by Ensor et al.^24^ who affirm that hospital budgets are not driven by any primary mechanisms or formulae (such as a system of costed norms) but, rather, by annual adjustments to historical levels of spending to take into account, for example, public sector wage increases or general inflation.

Participants verbalised that they had no idea of how the budget was prepared, approved and allocated. Participants in this study expressed the need for more inclusion and consultation in the budget process. Findings reveal that participants want to be consulted in establishing what budget amounts they require for the nursing section prior to the budget submission. Presently, they are not aware of the amounts they are allocated per department as the funds are all combined into one budget for the entire nursing section. Similar to these findings, Wentzel’s^25^ study found that the current trend in budgeting is to decentralise the budget process down to the point of service because those rendering the service are more aware of specific circumstances and conditions prevailing in the day-to-day operations of the organisation. The findings of the study indicated that most of the respondents were not conversant with the budget allocation and did not have sufficient knowledge of the clinic budget.^25^

Another common finding that emerged from participants’ accounts was the desire to have control over and manage their own budgets and departments as separate independent cost centres. This they felt would give them greater autonomy and control over their department’s finances and would enable them to purchase goods and services according to their own individual needs and according to their specifications. The findings on the need for greater involvement and control over the budget are similar to those of Doyle and Williamson^26^ whose study was conducted to identify the knowledge gap and learning needs of clinical or nurse managers posed by devolved budgeting within a changing environment.

### Theme 2: Financial monitoring

Further roles in financial management included income and expenditure monitoring and control and analysis of financial statements and reports. It was noted from the data gathered that the NM is responsible for the monitoring of expenditure and ensuring that this is within the allocated budget. The NM monitors orders and approves items ordered or requested. Part of the NM role as found in the study was also monitoring of payments. As revealed in the results, these functions were accomplished by the NM as part of the senior management team through participating in cash flow monitoring. These findings are in keeping with Cole-Ingait^27^ who explains that a health organisation generates transactions from its operational and strategic activities.

A second mechanism mentioned by participants as part of their financial monitoring role is the analysis and interpretation of financial statements. Booyens et al.^21^ state that the purpose of financial statements is to monitor how the institution spends income and what the current financial position is. A common finding that emerged in this study was that NMs found difficulty in understanding some of the financial terminology in the financial reports. These findings are supported by Finkler et al.^22^ who state that understanding financial statements is difficult, as the accounting terminology used is generally new to nurses. However, the authors are in strong support of nurses being able to understand these statements and find that basic accounting concepts are critical tools for NMs on two levels: NMs must be able to communicate with FMs of the organisation, and accounting provides information that is of critical value to NMs as they help manoeuvre the overall direction of the organisation. The need to acquire resources for nursing and to work with the organisation to control the use of resources requires the NM to be able to communicate with FMs or, at least to some extent, be able to talk some of their language.^22^

### Theme 3: Financial decision-making

Booyens et al.^21^ find that nurses are involved in complex decision-making, in diversity of situations and for many different purposes. The decisions that nurses make in their daily duties include those related to financial management decisions. The NMs identified that their decision-making, especially in financial management, was influenced by the financial legislative framework that sets out the guidelines on how each financial activity should be carried out. Part of the financial decision-making role also included the involvement in the awarding of contracts and tenders. This was also guided by the directives and legislation relevant to these specific activities.

Emerging from the context of decision-making, the findings reveal that this role of the NMs encompasses their involvement in bid committees where decisions are made in terms of how contracts and tenders are awarded. The findings also indicate that the NM makes decisions for all expenditure for the nursing section. The NM is part of the Cash Flow Committee which is where financial decisions are taken in terms of what needs to be procured and what must be purchased according to priority. Sunderland^28^ supports these findings as she highlights the value of NMs being decision-makers for procurement. The author finds that the NMs position on the front line of patient care means nurses are uniquely positioned to offer feedback on which products are best from a patient experience perspective. In so doing, clinical risk is reduced; patient experience and safety is improved; product standardisation becomes much easier, leading to economies of scale; and the procurement process becomes more efficient and effective.^28^

The findings reveal that in the absence of the CEO, NMs are called to assume the role and this involves a large amount of decision-making which often has financial implications. Nurse managers indicated that they act in the role of the CEO but voiced concern as they felt that they had to make financial decisions with little understanding of such subjects. The results reveal that there was a difference in the financial knowledge of the CEOs compared to the NMs, which was mainly attributed to the lack of training in financial matters and the poor inclusion of NMs in financial activities. In a similar finding, Magrath^29^ states that nurses bring a wealth of clinical understanding to the chief executive role, but they have to master business skills and a wider focus if they want to succeed.

### Theme 4: Financial control

It was also noted in the study that the nurses, being the largest workforce in the hospital, play a critical role in expenditure control.^9^

The NM’s role is to investigate and find reasons for the rise in costs. Asset and resource control was seen to be a large part of the role of all participants in this study who emphasised that it is their responsibility to carefully supervise and control the use of the resources. Resources include the management of physical resources, equipment, facilities and infrastructure. Muller et al.^18^ confirms this statement by explaining that an organisation’s physical resources are its tangible assets such as equipment, buildings, vehicles, stock and supplies. Information received from the participants reflected that they maintain ultimate responsibility for resources and assets regarding control and care of equipment. Booyens et al.^21^ observe that the NM’s responsibility is to ensure that all health care professionals are taught about the use of the equipment if they are not familiar with the equipment. If they do not know how to use the equipment, it can lead to inefficient and dangerous practices. The environment in which staff members work must be conducive to health care delivery. The team is accountable and responsible for the management of the physical and financial resources.^21^

Muller et al.^18^ assert that expenditure control is a dimension of financial management and relates to the implementation and control of the approved budget. The NM’s role is to control expenditure in the hospital. It was reported by participants that they maintained accountability and responsibility for expenditure control in their departments. Sherman and Bishop^30^ explain that after a unit budget is approved, it must be monitored continuously to ensure expenses stay within projected budgetary limits. The NM gets feedback on actual expenses; data that show any discrepancies between budgetary projections and actual results are called variances.

The FMs in this study gave a divergent version of how they perceived expenditure control in the hospitals. According to the data gathered from the interviews in this study, FMs perceived that nurses do not understand expenditure control processes and that they lack financial consciousness. It was further found that nurses attribute their lack of expenditure control to the fact that they have a big workload. Penner^9^ adds that nurses are often completely unaware of the costs of care in their in-patient or out-patient settings. Few staff nurses have any background or education in health care finance and often resist the idea that they need to think about the cost of nursing care. However, in these times of rapid change and ever more scarce resources, it is time for nurses to realise that their performance affects not only their patients’ health but also the financial health of their institution.^9^ The financial control function of NMs included the assets and resources control, expenditure control and cost centre management. Despite their years of experience and regular performance of the activities, NMs still found difficulty in understanding these functions and indicated the need for more training.

Relevant to this study, every decision made irrespective of the context has a financial connotation to it. Nurses make decisions daily with respect to the delivery of the best and quality care for their patients. This in turn results in the patient recovering and returning to society, employment and communities and leading a productive life. This also results in the prevention of litigation which consumes a large part of the budget with the consequences of lesser funds being available for essential resources required for delivery of care.

The findings of this study differ from other studies on financial management for NMs in the sense that they do not suggest that NMs must learn all the finer details of financial management. Nurse managers must be able to apply financial management concepts to NM practice. The NM does not need to move into the CFO’s office, but needs to be able to understand the relevant aspects of financial management applicable to practice. If he or she is required to practice financial management, then he or she needs to have the requisite knowledge and skills to do so.

## Strengths and limitations

One limitation was the small sample size of NMs. A further limitation was that interviews were the only method of data collection. Given the limited sample size, the findings noted in this study may not fully reflect the views of other NMs who are employed in the public sector. A potential to overcome this would have been to consider using an additional method of observing NMs in their daily tasks and role in their own work environment. Some participants gave inadequate information to some of the questions, which affected the strength of the analysis. The study was limited to one province only. Perceptions about the NM financial management role, competencies and financial management developmental needs could be different in other provinces and countries. The study was also conducted in the public hospitals and not extended to private hospitals and other health sectors.

## Implications or recommendations

The importance of financial knowledge and financial literacy for NMs has not been given much attention, and the important role that NMs play in the finances of the hospitals has been overlooked. Nurse managers are mandated to become proficient in financial management which is frequently beyond their education and experience. To address these issues and to support NMs, more formal financial education and training programmes are needed, and continuous development strategies should be developed to increase their skills. Also the Department of Health does not play its part in assisting the NMs in the financial management role as it largely overlooks the significant role of NMs in the hospitals and as part of the senior management team.

All departments should be run as cost centres. This gives the NMs authority over the budget and expenditure and will increase their accountability, especially regarding managing the finances of their departments. It is recommended that the current promotion criteria for NMs be reviewed against the changing requirements of the NM role. There must be an induction and orientation programme tailored to meet the financial management needs of NMs on promotion. It is recommended that there be an interdisciplinary approach when it comes to teaching finance to NMs. All curricula including basic, undergraduate, post-basic and postgraduate programmes must be reviewed to reflect a realistic view of the expected competencies of NMs rather than based on clinical outcomes. Financial management programmes must be simple and avoid the financial language that would scare away the NM. A competency framework should be developed to improve the financial management competencies of NMs.

## Conclusion

The intention of this study was to bring to light the fact that NMs play a very big part in the financial management of the organisations. It was clear from the literature sources that the NMs lack financial management skills, competencies and knowledge and that more training and education is required. It is important that the NMs understand financial management concepts.
